# Isolation of human TRPA1 channel from transfected HEK293 cells and identification of alkylation sites after sulfur mustard exposure

**DOI:** 10.1007/s00204-022-03411-1

**Published:** 2022-11-12

**Authors:** Katharina Müller-Dott, Horst Thiermann, Harald John, Dirk Steinritz

**Affiliations:** 1 Bundeswehr Institute of Pharmacology and Toxicology, Neuherbergstr. 11, 80937 Munich, Germany; 2 Walther-Straub-Institute of Pharmacology and Toxicology, Ludwig-Maximilians-University, 80336 Munich, Germany

**Keywords:** Agonists of hTRPA1, Amino acid modifications, HETE, Hydroxyethylthioethyl-moiety, Immunomagnetic separation, μLC-ESI MS/HR MS

## Abstract

Transient receptor potential (TRP) channels are important in the sensing of pain and other stimuli. They may be triggered by electrophilic agonists after covalent modification of certain cysteine residues. Sulfur mustard (SM) is a banned chemical warfare agent and its reactivity is also based on an electrophilic intermediate. The activation of human TRP ankyrin 1 (hTRPA1) channels by SM has already been documented, however, the mechanism of action is not known in detail. The aim of this work was to purify hTRPA1 channel from overexpressing HEK293 cells for identification of SM-induced alkylation sites. To confirm hTRPA1 isolation, Western blot analysis was performed showing a characteristic double band at 125 kDa. Immunomagnetic separation was carried out using either an anti-His-tag or an anti-hTRPA1 antibody to isolate hTRPA1 from lysates of transfected HEK293 cells. The identity of the channel was confirmed by micro liquid chromatography-electrospray ionization high-resolution tandem-mass spectrometry. Following SM exposure, hTRPA1 channel modifications were found at Cys^462^ and Cys^665^, as well as at Asp^339^ and Glu^341^ described herein for the first time. Since Cys^665^ is a well-known target of hTRPA1 agonists and is involved in hTRPA1 activation, SM-induced modifications of cysteine, as well as aspartic acid and glutamic acid residues may play a role in hTRPA1 activation. Considering hTRPA1 as a target of other SM-related chemical warfare agents, analogous adducts may be predicted and identified applying the analytical approach described herein.

**Supplementary Information:**

The online version contains supplementary material available at 10.1007/s00204-022-03411-1.

## Introduction

Transient receptor potential (TRP) channels are cation permeable channels that are responsible for both mechanical- and chemo-sensation. They are composed of six transmembrane domains with a pore loop between domain five and six and intracellular N- and C-termini (Story et al. 2003; Nilius et al. 2012; Paulsen et al. 2015). The TRP ankyrin 1 (TRPA1) channel is the only mammalian member of the TRPA group. One characteristic of human TRPA1 (hTRPA1) channels is its ankyrin repeat sequence located at the N-terminus. hTRPA1 is expressed by primary afferent nociceptors but is also found in non-neuronal tissues and cells including skin, skeletal muscle, lung and digestive organs (Büch et al. 2013; Steinritz et al. 2018; Meents et al. 2019).

hTRPA1 channels are triggered by multiple stimuli such as temperature (Bandell et al. 2004; Andersson et al. 2008), mechanical stress (Kwan et al. 2006), hypoxia, reactive oxygen species (Koivisto and Pertovaara 2015), endogenous compounds linked with tissue injury (Bautista et al. 2013) and reactive chemicals. The latter group includes different substances such as ilicin (Story et al. 2003), isothiocyanates (e.g., allyl isothiocyanate, AITC) (Bandell et al. 2004; Jordt et al. 2004; Hinman et al. 2006), garlic (Bautista et al. 2005; Macpherson et al. 2005), tetrahydrocannabinol (THC) (Jordt et al. 2004), cinnamon (cinnamaldehyde) (Bandell et al. 2004; Macpherson et al. 2006) and acrolein (Bautista et al. 2006).

Most activators are electrophiles which were found to covalently modify certain amino acid residues in the ankyrin region including especially the cysteine residues Cys^621^, Cys^641^ and Cys^665^ and the lysine residues Lys^620^ and Lys^710^ (Hinman et al. 2006; Bahia et al. 2016). In addition to cysteine modifications, disulfide bridges between cysteines also appear to play a role in hTRPA1 activation and therefore have an impact on the functional conformation of the channel (Hinman et al. 2006; Macpherson et al. 2007; Takahashi et al. 2008; Fischer et al. 2010; Wang et al. 2012; Paulsen et al. 2015; Bahia et al. 2016; Meents et al. 2019; Suo et al. 2019; Talavera et al. 2020). Covalent modifications of the referred cysteine residues were shown to be involved in hTRPA1 activation (Hinman et al. 2006; Macpherson et al. 2007; Sadofsky et al. 2011; Bahia et al. 2016). Accordingly, Stenger et al. (2015) demonstrated hTRPA1 activation by the alkylating chemical warfare agent sulfur mustard (SM) and its analog 2-chloroethyl ethyl sulfide (CEES) but the relevant mechanism still remained unknown.

SM is a chemical warfare agent and was first used during World War I (Paromov et al. 2007; Ghabili et al. 2011; Rose et al. 2018). Even though the use of SM is prohibited under the Chemical Weapons Convention (CWC), which is supervised by the Organisation for the Prohibition of Chemical Weapons (OPCW), SM has been used for chemical attacks in Syria since 2013 by the terrorist group known as "Islamic State" (John et al. 2019; Sezigen et al. 2019). SM alkylates a wide range of biomolecules such as DNA, RNA and proteins (Ludlum et al. 1994; Shakarjian et al. 2009) thereby causing its toxic effects on skin, eyes and the respiratory system (Kehe et al. 2008; Ghabili et al. 2011; Rose et al. 2018; Müller-Dott et al. 2020). Since SM was already shown to alkylate cysteine residues by adding a hydroxyethylthioethyl (*HETE*) moiety to a multitude of proteins including albumin, creatine kinase, α1-antitrypsin and transthyretin (Lüling et al. 2018, 2021; John et al. 2019; Schmeißer et al. 2022), it is conceivable that the alkylation of intracellular cysteine residues in the ankyrin region of hTRPA1 is also linked to ThRPA1 activation. This mechanism has already been demonstrated for other reactive compounds such as cinnamaldehyde, iodoacetamide (IAA) and AITC (Hinman et al. 2006; Macpherson et al. 2007; Paulsen et al. 2015). Whether SM as a highly electrophilic molecule alkylates amino acid residues in hTRPA1 was investigated in the present in vitro study providing insights into hTRPA1 activation by SM. Therefore, hTRPA1 overexpressing HEK293 cells were used and hTRPA1 expression was monitored by Western blot analysis. Immunomagnetic separation (IMS) was used to extract the channel, followed by micro liquid chromatography-electrospray ionization high-resolution tandem-mass spectrometry (μLC-ESI MS/HR MS) to detect and identify SM-induced hTRPA1 channel alkylation sites.

## Materials and methods

### Chemicals

Dulbecco’s modified eagle medium (DMEM), fetal calf serum (FCS), penicillin/streptomycin solution (P/S), 0.05% trypsin–EDTA and phosphate-buffered saline (PBS) were purchased from Gibco by Life Technologies (Karlsruhe, Germany). PromoFectin transfection reagent was obtained from PromoCell GmbH (Heidelberg, Germany). The DNA construct pcDNA3.1V5-HisB_A123 and the HisPur™ Ni–NTA Spin Purification kit were purchased from ThermoFisher Scientific (Darmstadt. Germany). NuPAGE MES SDS running buffer (20x), NuPAGE transfer buffer (20x), 4–12% Bis–Tris gels, polyvinylidene difluoride (PVDF) membranes (0.2 µm pore size) and Dynabeads protein G were purchased from Invitrogen by Life Technologies (Karlsruhe, Germany). Digitionin, tris(2-carboxyethyl)phosphine-hydrochloride (TCEP-HCl), NaCl, dimethyl-pimelimidate-dihydrochloride (DMP), Tween-20, IAA, NH_4_HCO_3_, acetonitrile, triethanolamine (TEA), NaN_3_, trypsin and the corresponding trypsin reaction buffer from the Trypsin Profile IGD kit were obtained from Sigma-Aldrich (Steinheim, Germany). Tris, acetic acid, formic acid (FA ≥ 98%) and NaOCl solution for decontamination (12% Cl_2_) were obtained from Carl Roth (Karlsruhe, Germany). Threefold deuterated atropine (d_3_-atr) was from CDN Isotopes (Pointe Claire, Quebec, Canada). Chameleon Duo marker, 4 × protein loading dye, intercept blocking buffer PBS and IR dye 800CW goat anti-mouse antibody were obtained from Licor (Bad Homburg, Germany). PhastGel Blue tablets were from GE Healthcare (Munich, Germany). Methanol was purchased from Merck (Darmstadt, Germany). 1,4-dithiothreitol (DTT) was purchased from Roche (Penzberg, Germany). The primary anit-hTRPA1 antibody ANKTM-1 (C-5) was obtained from Santa-Cruz Biotechnology (Heidelberg, Germany) and anti-6xHis antibody from abcam (Cambridge, UK). SM (purity and integrity were assessed in-house by nuclear magnetic resonance, NMR, spectroscopy) was made available by the German Ministry of Defense.

### Cell culture

Human embryonic kidney wildtype (HEK-wt) cells, kindly donated by the Walther-Straub-Institute (Ludwig-Maximilians-University, Munich), were cultured in DMEM containing 10% (*v*/*v*) FCS and 1% (*v*/*v*) P/S in a humidified atmosphere at 37 °C, 5% (*v*/*v*) CO_2_. For transfection, 4–5 × 10^6 ^HEK-wt cells were seeded in a T175 flask. The next day, cells reached approx. 50% confluency and were transfected with pcDNA3.1V5-HisB_A123 using PromoFectin as follows: 10 µg of the DNA construct and 20 µL PromoFectin solution were each mixed with 1 mL DMEM without supplements. Afterwards, 1 mL PromoFectin solution was added to 1 mL DNA solution. The DNA-PromoFectin mix was incubated for 20 min at room temperature (RT). After removal of the cell culture medium, 2 mL of PromoFectin-DNA solution was added to the flask and filled up with 15 mL DMEM and incubated for 72 h. Transfected and thus hTRPA1 overexpressing HEK293 cells are further referred to as HEK-A1 cells.

### Cell lysis

A digitonin lysis buffer was used according to Suo et al. (2019). It was freshly prepared and contained 20 mM Tris, adjusted to pH 8.0, 150 mM NaCl, 5 mM TCEP-HCl and 1% (*w*/*v*) digitonin. Protease inhibitors and DNase were not part of the lysis buffer. For cell lysis, 2 mL lysis buffer was added to each T175 flask. Cells were incubated at 4 °C for 1 h before being gently detached by scraping. The whole content was transferred to another Eppendorf tube. The mixture was further incubated on ice for 1 h and intermittently vortexed. The mixture was centrifuged at 14,000 RCF for 15 min and supernatants were stored at − 80 °C.

### Immobilized metal affinity chromatography 

Cell lysates were purified using immobilized metal affinity chromatography (IMAC). The HisPur™ Ni–NTA spin purification kit, containing 1 mL columns, was used according to the manufacturer’s protocol. In brief, the equilibration buffer contained 10 mM imidazole, the wash buffer 25 mM imidazole and the elution buffer 300 mM imidazole. All centrifugation steps were carried out at 700 RCF for 2 min at 4 °C. The column was equilibrated with 2 mL equilibration buffer at 4 °C for 1 h. The column was then centrifuged and the equilibration fraction was collected (F1). The column was washed four-times with 2 mL wash buffer each, centrifuged, and the wash fractions were collected in separate tubes (W1-W4). The His-tagged protein was eluted with two-times 1 mL elution buffer followed by centrifugation and collection of the eluates in separate vials (E1-E2). All samples were stored at − 80 °C.

### Sodium dodecyl sulfate–polyacrylamide gel electrophoresis

The loading buffer contained 60 µL of 4 × loading dye mixed with 40 µL DTT (500 mM). HEK-A1 whole cell lysate (15 µL) was mixed with 8 µL of the loading buffer and loaded onto a 4–12% Bis–Tris gel. As a marker, 3 µL Chameleon Duo marker was used. Sodium dodecyl sulfate–polyacrylamide gel electrophoresis (SDS-PAGE) was run in ice-cold 1 × NuPAGE MES SDS running buffer at constant voltage (200 V) for 40 min. The gel was either stained with Coomassie brillant blue (CBB) or Western blotting was performed.

### Staining of proteins

Proteins were visualized in gel using CBB. A CBB stock solution was composed of one PhastGel Blue tablet dissolved in 200 mL H_2_O/methanol (40:60 *v*/*v*). For protein staining, CBB working solution was freshly prepared and contained 6 mL methanol, 12 mL water, 2 mL acetic acid (100%) and 2.2 mL of CBB stock solution (0.2% *w*/*v*). After SDS-PAGE, the gel was carefully removed from the chamber and placed in the CBB solution. The gel was incubated for 45 min on a swirl plate until bands were noticeably stained. CBB solution was then removed and the gel was washed twice with water for 15 min followed by additional de-staining overnight in water. The next day, the protein double band considered as hTRPA1 channel was cut out and subjected to nano-LC MS/MS analysis for protein identification.

### Western blot analysis

After SDS-PAGE, proteins were transferred from the gel onto a 0.2 µm PVDF membrane using the wet blotting technique. The membrane was activated in methanol for 30 s and blotting was performed using 1 × transfer buffer containing 20% (*v*/*v*) methanol at 25 V for 1 h. The membrane was then blocked in PBS blocking buffer for 1 h on an orbital shaker. As primary antibody solution, either 4 µg anti-hTRPA1 (ANKTM1 C-5) or 4 µg anti-His-tag (anti-6xHis) antibody was diluted in 4 mL 0.2% (*v*/*v*) Tween-20 in PBS blocking buffer and incubated overnight. The membrane was washed twice for 10 min with a washing buffer containing 0.1% (*v*/*v*) Tween-20 in PBS. The commercial solution of the secondary antibody (800CW goat anti-mouse antibody) was diluted 1:7500 in 11 mL 0.2% (*v*/*v*) Tween-20 in PBS blocking buffer and the membrane was incubated for 1 h. Afterwards, the membrane was washed twice as described above. Images were recorded with an Odyssey^®^ DLx imaging system using the software Image studio 5.2 (Licor, Bad Homburg, Germany).

### Measurement of the intracellular calcium concentration with Fura-2 AM

Fura-2 AM was used according to the manufacturer’s instructions. A stock solution (1 mM) was prepared. Cells were harvested in the same manner as described before (see section: cell culture) and counted using a CASY Cell Counter and Analyzer TT. Approximately 2*10^6^ cells/mL was used and the cell suspension was centrifuged for 5 min at 500 RCF. Afterwards, cells were loaded with Fura-2 AM (c_final_ 2 µM) and incubated for 1 h at 37 °C. The cells were then centrifuged (500 RCF, 3 min), rinsed with DMEM, and centrifuged again (500 RCF, 3 min). Cells were resuspended in DMEM before plating 190 µL of cell suspension into each well of a black 96-well plate. In the case of AP18 pre-treatment, cells were resuspended in DMEM with AP18 (final concentrations: 5 μM, 10 μM and 25 μM). The photometer was adjusted to 37.0 °C. The injector was then primed with the before prepared AITC solution (1 mL). DMEM was used as negative control. The excitation wavelengths for Ca^2+^-bound and Ca^2+^-free fura-2 AM were set to 340 nm and 380 nm, respectively. The wavelength of maximum emission in both forms was 510 nm. The ratios 510 nm/340 nm and 510 nm/380 nm are proportional to the quantity of Ca^2+^ present intracellularly. After recording the baseline for 20 cycles (approx. 40 s), 10 μL of the agonist was injected and changes in fluorescence were measured for 150 s. A 340 nm to 380 nm ratio was determined to assess changes in intracellular calcium levels ([Ca^2+^]_*i*_).

### Immunomagnetic separation

Preparing IMS of hTRPA1, commercially available Dynabead protein G slurry (1 mL) was transferred into a 5 mL reaction vial. Beads were fixed using a magnet and the supernatant was discarded. Beads were washed three-times with 2 mL PBST (0.05% *v*/*v* Tween-20 in PBS, pH 7.4). Afterwards, 4 mL PBST was added to the beads and either 200 µL of anti-His-tag antibody (1 mg/mL) or 1 mL of anti-hTRPA1 antibody solution (200 µg/mL) was added for immobilization. The mixture was incubated on a rolling shaker for 15 min at RT. The supernatant was removed, and beads were washed twice with 200 mM TEA (0.025% *w*/*v* NaN_3_, pH 7.8). Afterwards, beads were incubated with a freshly prepared DMP solution (2 mL, 5.4 mg/mL in TEA solution) on a rolling shaker for 30 min at RT. The supernatant was again removed and 2 mL Tris-buffered saline (TBS) (0.9% *w*/*v* NaCl in 20 mM Tris, pH 7.6) was added. The mixture was incubated for 15 min at RT on a rolling shaker. The beads were washed twice with 1 mL PBST. Finally, 950 µL PBST was added to the labeled beads which were stored at 4 °C.

The labeled beads slurry (50 µL) was transferred into a 1.5 mL reaction vial. Beads were fixed with a magnet and the supernatant was discarded. Afterwards, 200 µL cell lysate was added and the mixture was incubated for 2 h at 20 °C. The supernatant was removed, and beads with the bound protein were washed twice with 500 µL PBST, each. Next, 200 µL PBS and 8 µL DTT (20 mg/mL in water) were added to the vial. Samples were incubated for 30 min at 47 °C. Afterwards, 16 µL IAA (40 mg/mL in water) was added following a 30 min incubation at RT in the dark. The protein labeled beads were washed three-times with 250 µL NH_4_HCO_3_ (4 mg/mL) and the supernatant was removed. For proteolysis, 25 µL trypsin solution (20 µg/mL in trypsin solubilization reagent) and 50 µL trypsin reaction buffer (40 mM NH_4_HCO_3_ in 9% *v*/*v* acetonitrile) was added and incubated overnight at 37 °C under gentle shaking. Afterwards, the supernatant was transferred into a 10 kDa ultrafiltration (UF) device for UF (10,000 RCF, 10 min, 15 °C). The retentate was washed twice by UF with 50 µL d_3_-atr solution (3 ng/mL in 0.5% *v*/*v* FA). The filtrates were centrifuged and subjected to μLC-ESI MS/HR MS analysis or stored at − 20 °C.

### Exposure of extracted hTRPA1 protein to SM

HEK-A1 cell lysate (200 µL) was incubated with antibody-labeled beads for 2 h for extraction of hTRPA1 by IMS. Beads with bound hTRPA1 were mixed with 195 µL PBS and incubated (1 h, RT) with 5 µL ethanolic SM solution (diluted in ethanol yielding final concentrations of 1 to 10 mM SM). Blanks had a final concentration of 2.5% (*v*/*v*) ethanol only. The liquid layer was discarded and IMS was performed as described above (see section: Immunomagnetic separation).

### Exposure of cell culture to SM

HEK-A1 cells were exposed to SM as follows: cell culture medium was removed from the T175 flask 72 h after transfection and 6 mL of an ethanolic SM solution diluted in DMEM (final concentrations: 50 µM, 100 µM, 200 µM, 250 µM, 500 µM and 1000 µM) was added to each flask. As a negative control (blank), 2.5% (*v*/*v*) ethanol was used. The cells were incubated for 1 h at 37 °C. Due to the detachment of the cells after exposure to SM concentrations above 500 µM, cell-containing supernatants were collected and centrifuged for 5 min at 500 RCF. The cell pellet was washed with PBS. Cells were again centrifuged and 2 mL lysis buffer (see section: Cell lysis) was added. The pellet was resuspended in lysis buffer and incubated for 1 h on ice. The lysate was further incubated for 1 h and vortexed several times. Cell lysates were centrifuged for 15 min at 14,000 RCF and the supernatants were subjected to clean tubes. Lysates were stored at − 80 °C.

### µLC-ESI MS/HR MS analysis

A MicroPro pump (Eldex Laboratories, Napa, CA, USA) with an Integrity autosampler and a Mistral column oven (both Spark Holland, Emmen, The Netherlands) were used for chromatography that was online coupled to a QExactive plus Orbitrap mass spectrometer by an HESI II ion source (Thermo Scientific, Bremen, Germany). Eldex MicroPro 1.0.54 software (Eldex Laboratories) was used to control the system (Blum et al. 2020; John et al. 2022a). The Excalibur 4.1 software (Thermo Scientific) was used to manage the MS system. Calibration was done daily using the Pierce LTQ Velos ESI positive ion calibration solution (Thermo Fisher Scientific). The lock masses of protonated ubiquitous molecules (C_24_H_39_O_4_, *m/z* 391.28429, and C_10_H_16_O_2_NS, *m/z* 214.08963) were used for internal mass calibration (John et al. 2022a). To identify peptides obtained from proteolysis of the adducted hTRPA1 channel initial MS/HR MS detection was carried out in the ddMS2 approach. Proteome Discoverer Software 2.5.0.400 (Thermo Scientific) was used to analyze the obtained data.

Using the ddMS2 approach, peptides were separated at 45 °C using a binary mobile phase (30 µL/min) of solvent A (0.05% *v*/*v* FA) and solvent B (ACN/H_2_O 80:20 *v*/*v*, 0.05% *v*/*v* FA) on an Acquity UPLC HSS T3 column (150 × 1.0 mm I.D., 1.8 μM, Waters, Eschborn, Germany) protected by a precolumn (Security Guard Ultra cartridge C18 peptide; Phenomenex, Aschaffenburg, Germany). Solvent A and solvent B were applied in gradient mode: *t*[min]/*B*[%] 0/4; 3/4; 60/40; 60.5/95; 68.5/95; 69/4; 70/4 with an initial 15 min equilibration period under starting conditions. Eluates between retention time (*t*_R_) 4.5 min and 60 min were directed toward the mass spectrometer using a six-port valve. For full scan MS analysis, resolution was 70,000 full-width at half-maximum (fwhm) (John et al. 2022a) and the scan range was from *m/z* 290 to *m/z* 2,000. The ddMS2 was recorded with a resolution of 17,500 fwhm and loop count was set to 10. A stepped normalized collision energy of 25 was chosen and *m/z* 100 was selected as fixed first mass. Analysis of the detected peptides was performed using the Proteome Discoverer software. All possible *HETE* modifications at e.g., cysteine, glutamic acid and aspartic acid residues were added to the inclusion list. Detailed settings are listed in supplementary information (Protocol SI 1).

For more sensitive and selective detection of identified modified peptides, the parallel reaction monitoring (PRM) mode was chosen as a second targeted approach and chromatography was carried out on an Acquity HSS T3 column (50 × 1 mm I.D. 1.8 μm, Waters) with solvent A and solvent B in gradient mode (30 μL/min, 40 °C, *t*[min]/*B*[%]: 0/2; 2.5/10; 3/20; 15/45; 16/98; 18/98; 19/2; 20/2, 10 min equilibration under starting conditions). Spray voltage was optimized to 3.0 kV. Eluates from *t*_R_ 0 min to 20 min were directed toward the mass spectrometer. First, full scan MS (resolution 70,000 fwhm, scan range *m/z* 100 to *m/z* 1,500) was carried out followed by PRM scans. Resolution was set to 17,500 fwhm and fixed first mass was *m/z* 100. PRM analyses were carried out for: GAKPC^192^(-*HETE*)K [M + 2H]^2+^ (*m/z* 354.18258); KGAKPC^192^(-*HETE*)KSNK [M + 2H]^2+^ (*m/z* 582.81502); WGC^199^(-*HETE*)FPIHQAAFSGSK M + 3H]^3+^ (*m/z* 580.60593); EC^213^(-*HETE*)MEIILR [M + 2H]^2+^ (*m/z* 555.77143); EC^213^(-*HETE*)MEIILR [M + 3H]^3+^ (*m/z* 370.85005); ID^339^(-*HETE*)SEGR [M + 2H]^2+^ and IDSE^341^(-*HETE*)GR [M + 2H]^2+^ (*m/z* 390.68145); INTC^462^(-*HETE*)QR [M + 2H]^2+^ (*m/z* 419.69912); WDEC^608^(-*HETE*)LK [M + 2H]^2+^ (*m/z* 449.19588) and YLQC^665^(-*HETE*)PLEFTK [M + 2H]^2+^ (*m/z* 673.33017). The FreeStyle 1.3 software was used for data processing (Thermo Scientific).

## Results and discussion

While the activation mechanism of hTRPA1 has been unraveled for some compounds including AITC (Jordt et al. 2004; Hinman et al. 2006; Macpherson et al. 2007), only little is known about SM. Therefore, we isolated the hTRPA1 channel from overexpressing HEK-A1 cells and used μLC-ESI MS/HR MS to identify SM-modifications that may cause hTRPA1 activation.

### Isolation and identification of hTRPA1 from overexpressing HEK-A1 cells

**Fig. 1:**
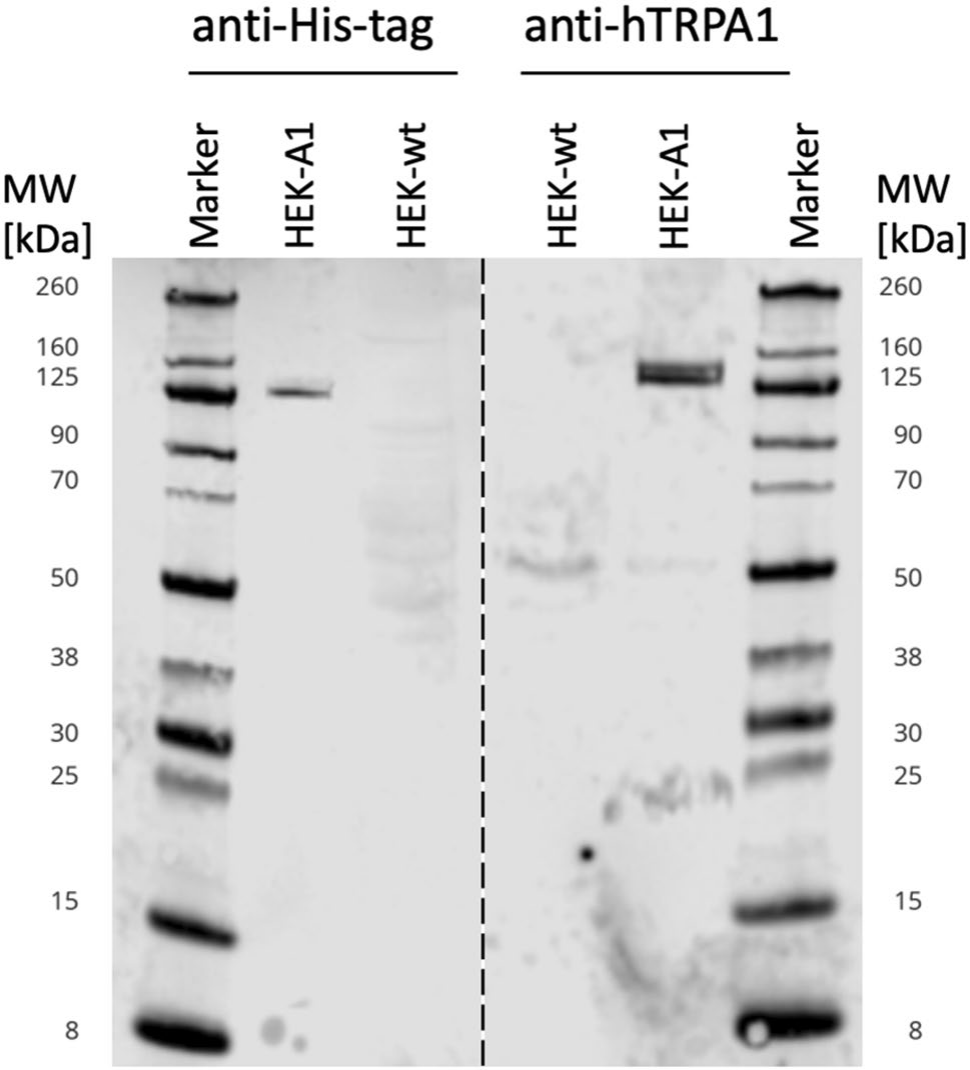
Western blot analysis of HEK-A1 and HEK-wt cell lysates. Anti-His-tag antibody (left) and anti-hTRPA1 antibody (right) were used for detection of hTRPA1. Lane 1 and 6: Chameleon Due marker, lane 2 and 5: total protein lysate of transfected HEK-A1 cells; lane 3 and 4: total protein lysate of HEK-wt cells (negative control). HEK-A1 protein lysates showed a double band at 125 kDa indicating hTRPA1 expression detected with both antibodies, whereas no hTRPA1 was found in HEK-wt cells

Successful expression of hTRPA1 was proven by Western blot analysis and Ca^2+^-measurements. As already discussed by Virk et al. (2019), the anti-hTRPA1 and the anti-His-tag antibody showed a distinct and characteristic double band at 125 kDa corresponding to the size of the hTRPA1 channel (Fig. 1, lane 2 and 5). The double band was presumably caused by different posttranslational modifications not specified in the literature so far (UniProt No O75762, https://www.uniprot.org/uniprotkb/O75762/entry). HEK-wt cells were used as negative control (blank) and thus, did not show any band (Fig. 1, lane 3 and 4).

To ascertain that these protein bands corresponded to the His-tagged hTRPA1 channel, it was purified from protein lysates using IMAC. Sequence analysis by MS/MS-based methods allowed identification of both protein bands as hTRPA1 (UniProt No O75762) with a Mascot probability score of 1165.

To prove expression in cell culture resulted in functional hTRPA1, changes in [Ca^2+^]_*i*_ were measured using Fura-2 AM (Almers and Neher 1985). HEK-A1 cells showed a concentration-dependent rise in [Ca^2+^]_*i*_ after injection of the agonist AITC (Fig. SI1A) (Bandell et al. 2004; Hinman et al. 2006). To confirm that the observed signals were mediated through hTRPA1, the specific antagonist AP18 was used to block hTRPA1. Changes in [Ca^2+^]_*i*_ were reduced and nearly disappeared when AP18 concentrations increased (Fig. SI1B). In addition, AITC exhibited no impact on HEK-wt cells (Fig. SI1A). Thus, expression of functional hTRPA1 in HEK-A1 cells was confirmed.

### Identification of alkylation sites

**Fig. 2:**
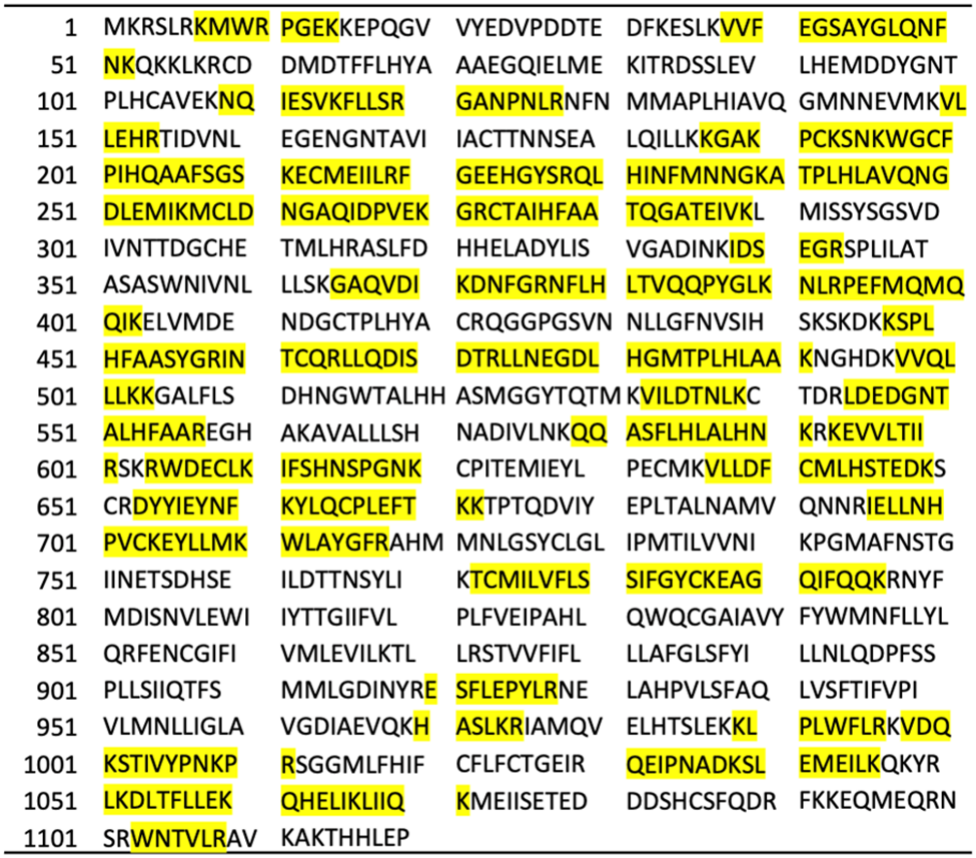
Amino acid sequence of hTRPA1 (UniProt No O75762). Peptides detected by µLC-ESI MS/HR MS obtained after tryptic cleavage of isolated not SM-exposed hTRPA1 are highlighted in yellow (sequence coverage 40%). Human TRPA1 was extracted by IMS using either an anti-His-tag or an anti-hTRPA1 antibody. Cysteine residues including Cys^192^, Cys^199^, Cys^213^, Cys^258^, Cys^273^, Cys^462^, Cys^608^, Cys^665^, Cys^703^, Cys^773^ and Cys^786^ were found to be carbamidomethylated

IMS, using either the anti-His-tag or the anti-hTRPA1 antibody, allowed the purification of hTRPA1 from total cell lysate and its identification by µLC-ESI MS/HR MS (ddMS2 approach, sequence coverage 40%, Mascot probability score of approx. 800). These results were obtained from lysates of different non-exposed cell passages showing the same peptide pattern after proteolysis. A number of peptides was detected and identified by ddMS2 analysis as highlighted in Fig. 2. Other proteins detected in the samples were immunoglobulin κ variable 2–40 (UniProt No A0A087WW87), 3–15 (UniProt No P01624), 4–1 (UniProt No P06312), immunoglobulin λ variable 8–61 (UniProt No A0A075B6I0) and also immunoglobulin heavy constant γ 2 (UniProt No P01859). Immunoglobulins obviously originated from the antibodies used in the IMS procedure.

SM is known to alkylate thiol groups of cysteine residues by attaching a *HETE*-moiety (Lüling et al. 2018, 2021; John et al. 2019; Schmeißer et al. 2022) as well as carboxy-groups of aspartic acid and glutamic acid residues (John et al. 2019, 2022b). Based on the peptide pattern detected from non-exposed hTRPA1, we calculated the masses of the protonated and potentially alkylated peptides for subsequent targeted μLC-ESI MS/HR MS (PRM) analysis of SM incubated samples. This procedure provided optimum selectivity and sensitivity to unravel modified peptides containing the *HETE*-moiety either as a thioether in cysteine residues (e.g., Cys^192^, Cys^199^, Cys^213^, Cys^258^, Cys^273^, Cys^46*2*^, Cys^608^, Cys^665^, Cys^703^, Cys^773^ and Cys^786^) or as an O-ester in Asp^339^ or Glu^341^. Except for Cys^665^, Cys^703^, Cys^773^ and Cys^786^, all targeted cysteine residues are located in the ankyrin region (Paulsen et al. 2015). In addition, Cys^665^ has already been identified to play a pivotal role in hTRPA1 activation (Bahia et al. 2016; Meents et al. 2019; Talavera et al. 2020), thus, these cysteine residues and especially Cys^665^ appear as potential targets also for alkylation by SM.

### Alkylation of hTRPA1 by SM

**Fig. 3:**
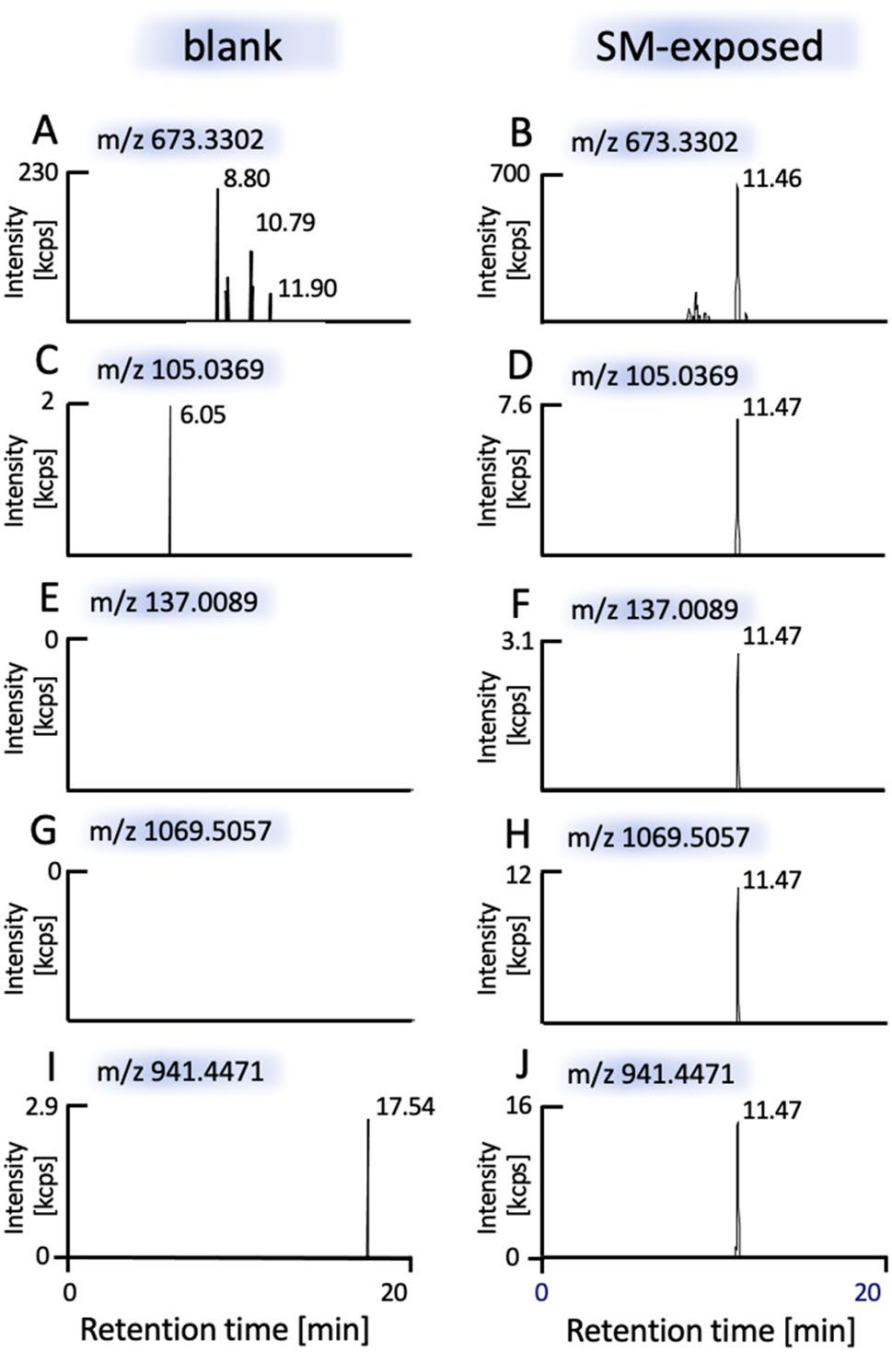
Detection of the alkylated decapeptide YLQC^665^(-*HETE*)PLEFTK [M + 2H]^2+^ using μLC-ESI MS/HR MS (PRM). Results from a blank (negative control) not exposed to SM are shown in left column (**A**, **C**, **E**, **G**, **I**) and results of HEK-A1 cells exposed to SM are shown in right column (**B**, **D**, **F**, **H**, **J**). Human TRPA1 was extracted from HEK-A1 cells by IMS and subjected to trypsin-mediated proteolysis. The XIC of the alkylated peptide ([M + 2H]^2+^ *m/z* 673.3302, ± 3 ppm) is shown in Fig. 3B and showed one peak at *t*_R_ 11.46 min. The XIC of diverse product ions (± 10 ppm) assigned in Table SI1 and Figure SI2 are shown in part D (*m/z* 105.0369), F (*m/z* 137.0089), H (*m/z* 1069.5057) and J (*m/z* 941.4471) and revealed one peak at t_R_ 11.47 min. No interferences were observed in the blank (**A**, **C**, **E**, **G**, **I**)

**Fig. 4:**
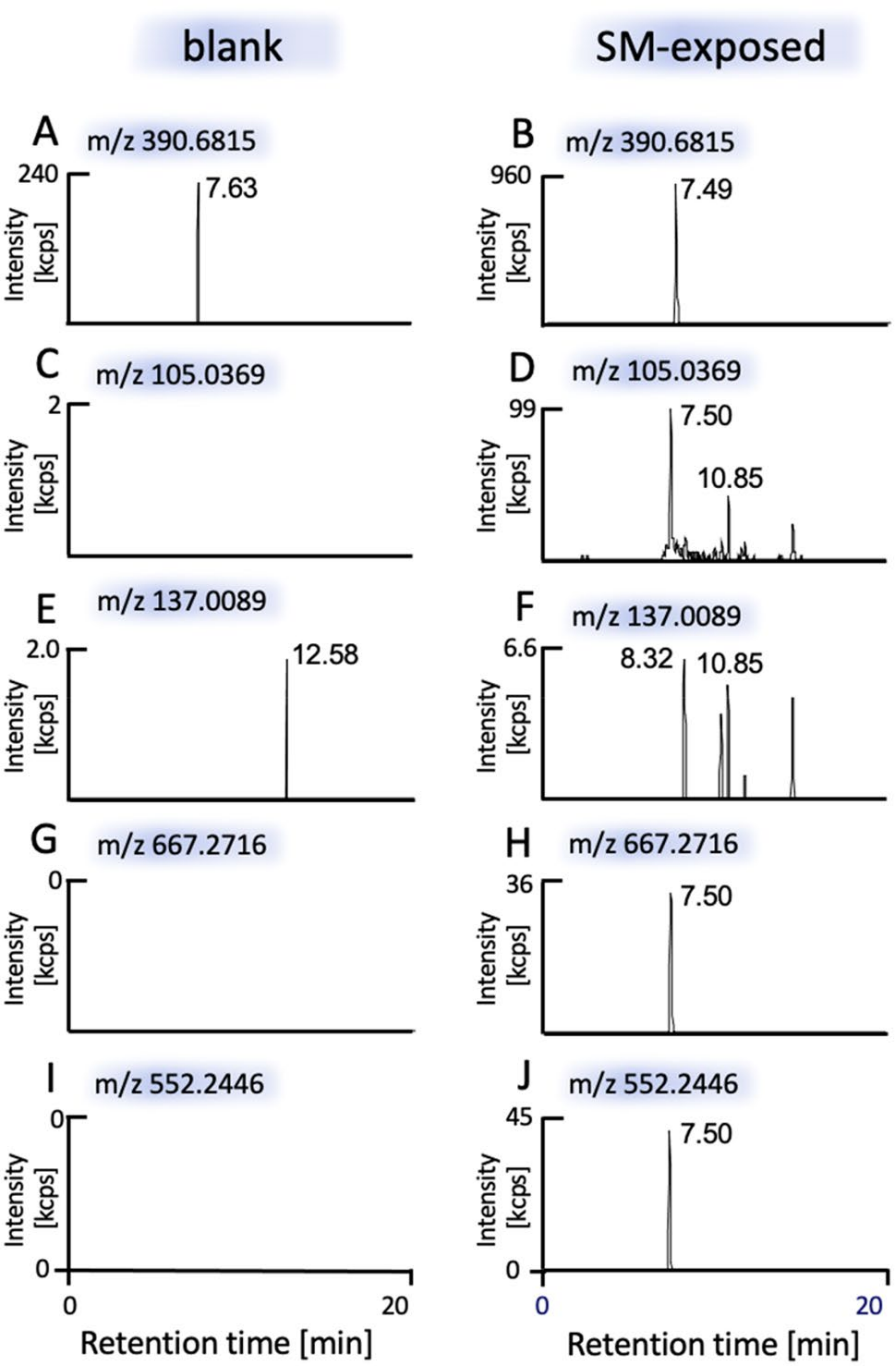
Detection of the alkylated hexapeptides ID^339^(-*HETE*)SEGR [M + 2H]^2+^ and IDSE^341^(-*HETE*)GR [M + 2H]^2+^ using targeted μLC-ESI MS/HR MS (PRM). Results from a blank (negative control) not exposed to SM are shown in left column (**A**, **C**, **E**, **G**, **I**) and results of HEK-A1 cells exposed to SM are shown in right column (**B**, **D**, **F**, **H**, **J**). Human TRPA1 was extracted from HEK-A1 cells by IMS and subjected to trypsin-mediated proteolysis. The XIC of the alkylated peptide ([M + 2H]^2+^, *m/z* 390.6815) is shown in Fig. 4B (± 3 ppm) and the XIC of diverse product ions (± 10 ppm) assigned in Table SI 3, SI 4 and Figure SI5 are shown in part **D** (*m/z* 105.0369), **H** (*m/z* 667.2716) and J (*m/z* 552.2446) and revealed one peak at *t*_R_ 7.50 min. The absence of an ion peak at the relevant *t*_R_ of the XIC of *m/z* 137.0089 (**F**) indicated that no cysteine residue was alkylated. No interferences were observed in the blank (**A**, **C**, **E**, **G**, **I**)

To identify alkylation sites, purified hTRPA1 from cell lysates was incubated with SM (10 mM). SM-induced hTRPA1 modifications were analyzed by μLC-ESI MS/HR MS (PRM). Four alkylated peptides were detected containing modified amino acids: INTC^462^(-*HETE*)QR (Fig. SI 3 and SI 4, Table SI 2), YLQC^665^(-*HETE*)PLEFTK (Fig. 3 and SI 2, Table SI 1) as well as ID^339^(-*HETE*)SEGR (Fig. 4 and SI5, Table SI3) and IDSE^341^(*-HETE*)GR (Fig. 4 and SI5, Table SI4).

After exposure of intact HEK-A1 cells to at least 250 μM SM, the extracted ion chromatogram (XIC) of full scan measurement of the double-protonated and alkylated peptide YLQC^665^(-*HETE*)PLEFTK (*m/z* 673.3302) showed a single peak at *t*_R_ 11.46 min (Fig. 3B). In PRM analysis of this peptide, the well-known diagnostic ions at *m/z* 105.0369, representing the *HETE*-moiety [*HETE*]^+^, and at *m/z* 137.0089, indicating the *HETE*-moiety with a sulfur atom of the cysteine residue [*HETE* + S]^+^, were also detected at *t*_R_ 11.47 min (Fig. 3D, F). Additionally, product ions at *m/z* 1069.5057 (y_8_ ion) and at *m/z* 941.4471 (y_7_ ion), both containing the *HETE*-moiety, were found at the same retention time. The peaks of the precursor ion as well as of the product ions were not present in the blank (HEK-A1 cells not exposed to SM, Fig. 3A, C, E, G, I) demonstrating the high selectivity and suitability of precursor and product ion detection. These results documented the unambiguous detection and identification of YLQC^665^(-*HETE*)PLEFTK containing the alkylated Cys^665^ residue. The MS/HR MS spectrum is shown in the supplement (Fig. SI2) and product ion assignment is summarized in Table SI1.

Following the same strategy, INTC^462^(-*HETE*)QR [M + 2H]^2+^ (containing alkylated Cys^462^) was also detected as a single peak in the XIC (*m/z* 419.6991) of a full scan analysis at *t*_R_ 7.28 min as well as single peak of diverse product ions from PRM analysis as illustrated in supplement Figs. SI3, SI4 and Table SI2. The absence of these product ions in the blank demonstrated the selectivity of the method and suitability of this peptide (Fig. SI3, left column).

In addition, we detected alkylation sites at the Asp^339^ and Glu^341^ residues after incubation of total protein lysate yielding the alkylated peptides ID^339^(-*HETE*)SEGR and IDSE^341^(*-HETE*)GR. A *t*_R_ of 7.49 min was observed for the precursor ion at *m/z* 390.6815 (Fig. 4B) as well as for the product ion at *m/z* 105.0369 (Fig. 4D) selectively indicating the presence of the *HETE*-moiety. No peak at the relevant *t*_R_ was detected at *m/z* 137.0089 (Fig. 4F) indicating that the alkylation site was not a cysteine residue. Diverse product ions containing the *HETE*-moiety were also detected as illustrated in Fig. 4H, J. Obviously both peptides ID^339^(-*HETE*)SEGR and IDSE^341^(*-HETE*)GR coeluted and were simultaneously subjected to fragmentation for MS/HR MS yielding a mixed product ion spectrum documenting that the *HETE*-moiety was attached to the Glu^341^ and also to the Asp^339^ residue as shown in Fig. SI5. The absence of the peaks of the precursor and product ions in the blank (Fig. 4A, C, E, G, I) proved the high selectivity and suitability of the detection.

We herein identified Cys^462^ and Cys^665^ as alkylation sites after SM exposure. In addition, we detected modifications at Asp^339^ and Glu^341^. Different mutation studies have previously revealed that Cys^414^, Cys^621^ and also Cys^665^ play an essential role in hTRPA1 activation by electrophiles (Hinman et al. 2006; Macpherson et al. 2007; Takahashi et al. 2008; Fischer et al. 2010; Bahia et al. 2016). Because Cys^665^ is located intracellularly in a flexible loop, it is solvent accessible and might be a target for reactive chemicals (Macpherson et al. 2007; Paulsen et al. 2015). This shows that also SM reacted intracellularly and was able to alkylate Cys^665^ as presented in this study. For other agonists, such as AITC and IAA, it was demonstrated that Cys^665^ was needed for electrophile-induced hTRPA1 activation (Macpherson et al. 2005; Bahia et al. 2016). Therefore, Cys^665^ might also be essential in SM-induced hTRPA1 activation. Additionally, four disulfide bridges, namely Cys^665^-Cys^621^, Cys^665^-Cys^462^, Cys^665^-Cys^192^ and Cys^621^-Cys^608^, were discovered in the absence of any reducing agent by Wang et al. (2012). These findings revealed that hTRPA1 channel activation involves conformational changes in the N-terminal region to provide accessibility of the cysteines (Wang et al. 2012). As Cys^665^ was identified as an alkylation site in our study, it is plausible that hTRPA1 conformation might be dynamically driven by electrophilic activation also by SM.

Cys^462^ is also located inside the ankyrin region in the cytoplasm (Paulsen et al. 2015), supporting that SM was able to act intracellularly. Without prior protein reduction, SM alkylated Cys^462^ which is in vivo disulfide-bridged with Cys^665^ (Wang et al. 2012) indicating that conformational changes within the hTRPA1 channel might occur after SM exposure. Furthermore, the SM concentration required for alkylation was two-times higher when compared to Cys^665^. Stenger et al. (2015) performed calcium measurements to evaluate hTRPA1 activation by SM, showing that 500 μM SM caused activation. Thus, we assume that Cys^462^ and Cys^665^ might be involved in hTRPA1 activation after SM exposure.

John et al. (2019, 2022b) showed that in addition to SM-induced modifications at cysteine residues, glutamic acid modifications were found in human and avian serum albumin. It is therefore plausible that alkylation at aspartic and glutamic acid in hTRPA1 had occurred. Both amino acids possess a free carboxylic group that can react with the electrophilic SM as already shown before (Smith et al. 2008; John et al. 2019, 2022b). Additionally, TRPC channels possess similar structures to hTRPA1 channels and have three to four ankyrin repeats at the N-terminus. These also contain glutamic acid residues that promote TRPC5 channel activation (Jung et al. 2003; Jiang et al. 2011). Thus, aspartic and glutamic acid modifications might also contribute to hTRPA1 activation. In the present study, modifications were only seen at quite high SM concentrations (≤ 1 mM SM) used for cell lysate incubation. A decreased in vivo stability of SM-induced protein modifications at aspartic or glutamic acid was demonstrated by Smith et al. (2008). Thus, the *HETE*-moiety might have been released from the protein and may explain the lack of alkylation following exposure of the intact cell system in our study. Various competitive reactions with other proteins as well as hydrolysis of SM prior to alkylation might have occurred and reduced the amount of reactive SM.

**Fig. 5:**
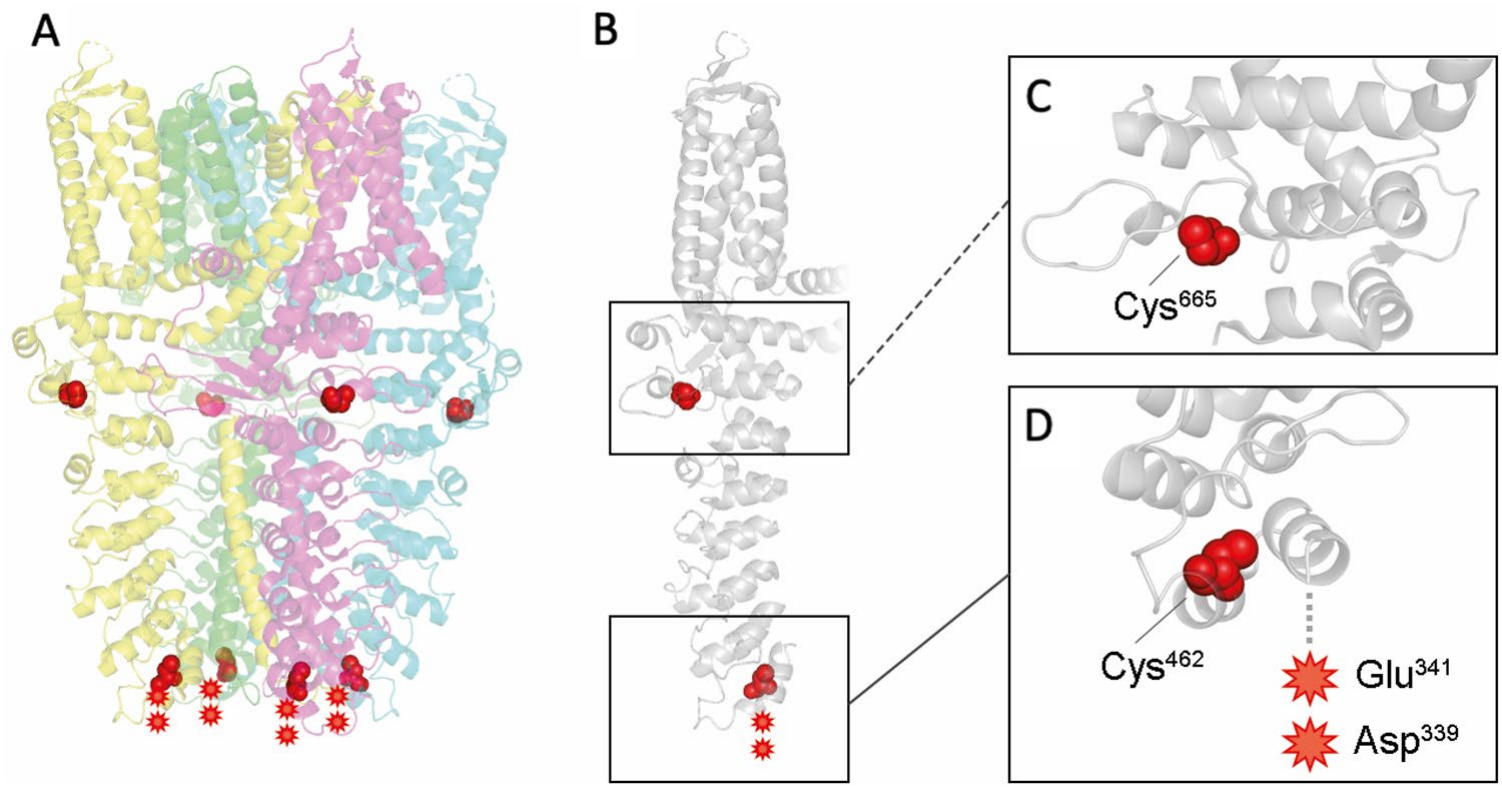
Structure of human TRPA1 protein with alkylation sites at Cys^462^, Cys^665^, Asp^339^ and Glu^341^. **A** The homotetrameric structure (PDB ID 6PQO) of hTRPA1 is shown with herein detected and described alkylation sites indicated in red. A monomeric structure (**B**) details the alkylation sites for Cys^665^ (**C**) and Cys^462^, Asp^339^ and Glu^341^ (**D**). The figure was created using PyMOL version 2.5.4

Additional cysteine residues (Cys^414^, Cys^421^, Cys^621^ and Cys^641^) have been reported in the literature to be significant for hTRPA1 activation (Hinman et al. 2006; Macpherson et al. 2007). Especially Cys^621^ has been described as a highly reactive hotspot for electrophile sensing (Bahia et al. 2016; Suo et al. 2019). Unfortunately, using the method described herein, peptide sequences covering Cys^414^, Cys^421^ as well as Cys^621^ were not detected. Accordingly, it remained unclear, whether these residues were targeted by SM. The two cysteines (Cys^462^ and Cys^665^) detected herein are structurally resolved and are shown in Fig. 5. Both cysteine residues are located in the cytosol and appear readily accessible for SM. The Asp^339^ and Glu^341^ are located at the N-terminal region (indicated with a red star Fig. 5D). The protein data bank entry (ID 6PQO) does not provide structural data on that region as it is characterized by a high flexibility. Access to any reactive side chain is therefore likely.

## Conclusion

We demonstrated for the first time that SM alkylates at least two cysteine residues (Cys^462^ and Cys^665^) as well as Asp^339^ and Glu^341^ in hTRPA1. Because hTRPA1 might be a target for additional chemical warfare agents structurally related to SM such as sesquimustard (Blum et al. 2020; Hemme et al. 2021) or O-mustard, similar adducts are expected and might be discovered using the analytical approach described herein. This approach might also be used for other TRP channels to elucidate potential binding sites. Future studies will address mutation experiments to assess the impact of the discovered alterations on functional activity of the channel. In this case, the modified cysteine residues as well as glutamic or aspartic acid will be altered to e.g., alanine and calcium measurements will be performed to determine the role of the mutated amino acid. This will provide further information on hTRPA1 activation and on specific amino acid residues which may directly be essential for hTRPA1 activation.

## Supplementary Information

Below is the link to the electronic supplementary material.

Supplementary file1 (PDF 758 KB)

## Abbreviations

AITC: Allyl isothiocyanate
[Ca^2+^]_i_: Intracellular calcium concentration
CBB: Coomassie brillant blue
CEES: 2-Chloroethyl ethyl sulfide
CWC: Chemical Weapons Convention
d_3_-atr: Triple deuterated atropine
DMEM: Dulbecco’s modified eagle medium
DMP: Dimethyl-pimelimidate-dihydrochloride
DTT: 1,4-Dithiothreitol
ESI: Electrospray ionization
FCS: Fetal calf serum
fwhm: Full-width at half-maximum
HEK-A1: HEK293 cells transfected and overexpressing His-tagged hTRPA1
HEK-wt: Human embryonic kidney 293 wildtype
*HETE*: Hydroxyethylthioethyl
hTRPA1: Human transient receptor potential ankyrin 1
IAA: Iodoacetamide
IMAC: Immobilized metal affinity chromatography
IMS: Immunomagnetic separation
MS/HR MS: High-resolution tandem-mass spectrometry
OPCW: Organisation for the Prohibition of Chemical Weapons
P/S: Penicillin/streptomycin solution
PBS: Phosphate-buffered saline
PRM: Parallel reaction monitoring
PVDF: Polyvinylidene difluoride
RT: Room temperature
SDS-PAGE: Sodium dodecyl sulfate–polyacrylamide gel electrophoresis
SM: Sulfur mustard
TBS: Tris-buffered saline
TCEP-HCl: Tris(2-carboxyethyl)phosphine-hydrochloride
TEA: Triethanolamine
THC: Tetrahydrocannabinol
*t*_R_: Retention time
TRP: Transient receptor potential
UF: Ultrafiltration
μLC: Micro liquid chromatography
