# Supplementary material for: Isolation of human TRPA1 channel from transfected HEK293 cells and identification of alkylation sites after sulfur mustard exposure
Source: Arch Toxicol. 2022 Nov 12;97(2):429–39. doi: 10.1007/s00204-022-03411-1 (PMC9859856; doi:10.1007/s00204-022-03411-1)
Supplement: Supplementary file 1 — Supplementary file1 (PDF 758 KB) [file 204_2022_3411_MOESM1_ESM.pdf]

Supplementary Information

ARCHIVES OF TOXICOLOGY

Isolation of human TRPA1 channel from transfected HEK293 cells and identification of alkylation sites after sulfur mustard exposure

Katharina Müller-Dott[1,2], Horst Thiermann[1], Harald John[1], Dirk Steinritz*[1]

[1] Bundeswehr Institute of Pharmacology and Toxicology, 80937 Munich, Germany

[2] Walther-Straub-Institute of Pharmacology and Toxicology, Ludwig-Maximilians-University, 80336 Munich, Germany

* Address correspondence to this author

Bundeswehr Institute of Pharmacology and Toxicology,

Neuherbergstr. 11, 80937 Munich, Germany

E-Mail: dirksteinritz@bundeswehr.org

Table of Content

Protocol SI 1: Settings for Proteome Discoverer software

Table SI 1: Precursor and product ions of YLQC^{665}(-*HETE*)PLEFTK

Table SI 2: Precursor and product ions of INTC^{462}(-HETE)QR

Table SI 3: Precursor and product ions of ID^{339}(-HETE)SEGR

Table SI 4: Precursor and product ions of IDSE^{341}(-HETE)GR

Figure SI 1: Changes in intracellular calcium concentration after stimulation with AITC using Fura-2 AM.

Figure SI 2: MS/HR MS spectrum of YLQC^{665}(-*HETE*)PLEFTK containing the alkylated Cys^{665} residue

Figure SI 3: Detection of hexapeptide INTC^{462}(-*HETE*)QR $[M+2H]^{2+}$ using targeted μLC-ESI MS/HR MS (PRM)

Figure SI 4: MS/HR MS spectrum of INTC^{462}(-*HETE*)QR containing the alkylated Cys^{462}

Figure SI 5: Mixed MS/HR MS spectrum of ID^{339}(-*HETE*)SEGR containing the alkylated Asp^{339} and of IDSE^{341}(-*HETE*)GR containing the alkylated Glu^{341}

Protocol SI 1

Protocol SI 1: Settings for Proteome Discoverer software

The following settings were applied: lowest charge was 1 and the highest charge was 6. As input data, human TRPA1 fasta file (UniProt No O75762) was selected and trypsin was selected as enzyme. Maximum number of missed cleavages was set to 3 and minimum peptide length was 4 amino acids, maximum peptide length was set to 30. Precursor mass tolerance was 5 ppm and fragment mass tolerance was set to 0.05 Da. Dynamic modifications included carbamidomethylation at cysteine and SM-modification at cysteine, glutamic acid, aspartic acid, histidine, lysine, methionine, arginine and tryptophan residues. Additionally, peptide spectral match (PSM) validator was added to the workflow. To determine Mascot probability score, the same settings as described before were used. Furthermore, as protein database SwissProt was selected and for taxonomy homo sapiens (human) was chosen.

Table SI 1

Table SI 1: Precursor and product ions of YLQC^{665}(*-HETE*)PLEFTK

ion	elemental composition	*m/z* theoretical (measured)	Δ*m/z* [ppm]
$[M+2H]^{2+}$	$C_{62}H_{98}N_{12}O_{17}S_2$	673.3302 (673.3300)	-0.3
y_1	$C_6H_{15}N_2O_2$	147.1128 (147.1126)	-1.4
y_2	$C_{10}H_{22}N_3O_4$	248.1605 (248.1603)	-0.7
y_3	$C_{19}H_{31}N_4O_5$	395.2289 (395.2285)	-1.0
y_4	$C_{24}H_{38}N_5O_8$	524.2715 (524.2718)	0.6
y_6	$C_{35}H_{56}N_7O_{10}$	734.4083 (734.4067)	-2.2
y_7	$C_{42}H_{69}N_8O_{12}S_2$	941.4471 (941.4399)	-7.6
y_8	$C_{47}H_{77}N_{10}O_{14}S_2$	1069.5057 (1069.4933)	-11.6
a_1	$C_8H_{10}NO$	136.0757 (136.0755)	-1.4
a_2	$C_{14}H_{21}N_2O_2$	249.1598 (249.1593)	-1.8
b_2	$C_{15}H_{21}N_2O_3$	277.1547 (277.1546)	-0.3
b_3	$C_{20}H_{29}N_4O_5$	405.2132 (405.2136)	-0.9
$y_8\text{-}18^{2+}$	$C_{47}H_{75}N_{10}O_{13}S_2$	526.2512 (526.2497)	-2.8
a_8^{2+}	$C_{51}H_{77}N_9O_{12}S_2$	535.7561 (535.7614)	9.9
$y_{10}\text{-}17^{2+}$	$C_{62}H_{95}N_{11}O_{17}S_2$	664.8169 (664.8205)	5.4

Table SI 2

Table SI 2: Precursor and product ions of INTC^{462}(-*HETE*)QR

ion	elemental composition	*m/z* theoretical (measured)	Δ*m/z* [ppm]
**$[M+2H]^{2+}$**	$C_{32}H_{61}N_{11}O_{11}S_2$	419.6991 (419.6981)	-2.4
**y_1**	$C_6H_{15}N_4O_2$	175.1189 (175.1182)	-4.3
**y_2**	$C_{11}H_{23}N_6O_4$	303.1775 (303.1779)	1.2
**y_3**	$C_{18}H_{36}N_7O_6S_2$	510.2163 (510.2147)	-3.1
**y_4**	$C_{22}H_{43}N_8O_8S_2$	611.2640 (611.2619)	-3.4
**y_5**	$C_{26}H_{49}N_{10}O_{10}S_2$	725.3070 (725.3035)	-4.7
**y_2 -17**	$C_{11}H_{20}N_5O_4$	286.1510 (286.1495)	-5.2
**y_5 -17**	$C_{26}H_{46}N_9O_{10}S_2$	708.2803 (708.2782)	-3.05
**y_5-18**	$C_{26}H_{47}N_{10}O_9S_2$	707.2963 (707.2949)	-2.0
**a_2**	$C_9H_{18}N_3O_2$	200.1394 (200.1386)	-3.8
**y_6 -17^{2+}**	$C_{32}H_{59}N_{10}O_{11}S_2$	411.1859 (411.1889)	7.4
**y_6 -18^{2+}**	$C_{32}H_{60}N_{11}O_{10}S_2$	410.6938 (410.6929)	-2.3
**a_6^{2+}**	$C_{31}H_{59}N_{11}O_9S_2$	396.6964 (396.6956)	-2.0
**b_6^{2+}**	$C_{32}H_{59}N_{11}O_{10}S_2$	410.6938 (410.6929)	-2.3

Table SI 3

Table SI 3: Precursor and product ions of ID[339](-*HETE*)SEGR

ion	elemental composition	*m/z* theoretical (measured)	$\Delta m/z$ [ppm]
**$[M+2H]^{2+}$**	$C_{30}H_{55}N_9O_{13}S$	390.6815 (390.6805)	-2.4
**y_1**	$C_6H_{15}N_4O_2$	175.1190 (175.1188)	-0.9
**y_4**	$C_{16}H_{30}N_7O_8$	448.2150 (448.2141)	-2.1
**y_5**	$C_{24}H_{43}N_8O_{12}S$	667.2716 (667.2703)	-1.9

Table SI 4

Table SI 4: Precursor and product ions of IDSE[341](-*HETE*)GR

Ion	elemental composition	*m/z* theoretical (measured)	Δ*m/z* [ppm]
**$[M+2H]^{2+}$**	$C_{30}H_{55}N_9O_{13}S$	390.6815 (390.6805)	-2.4
**y_1**	$C_6H_{15}N_4O_2$	175.1190 (175.1188)	-0.9
**y_4**	$C_{20}H_{38}N_7O_9S$	552.2446 (552.2425)	-3.9
**y_5**	$C_{24}H_{43}N_8O_{12}S$	667.2715 (667.2703)	-1.9
**a_2**	$C_9H_{17}N_2O_3$	201.1234 (201.1229)	-2.3
**a_2-18**	$C_9H_{15}N_2O$	183.1128 (183.1125)	-1.7
**b_2**	$C_{10}H_{17}N_2O_4$	229.1183 (229.1181)	-0.8

Figure SI 1

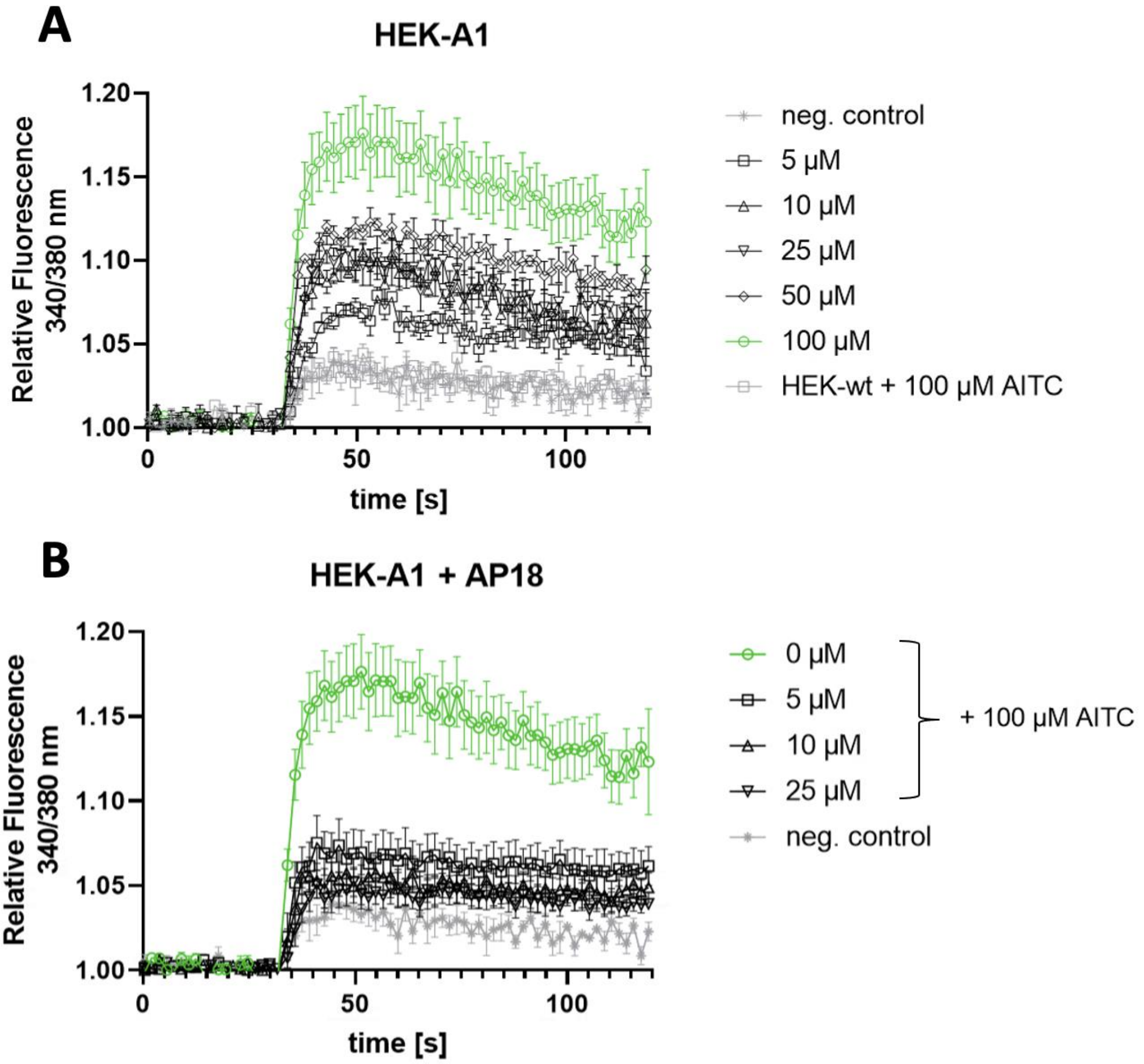

Figure SI 1: Changes in intracellular calcium concentration after stimulation with AITC detected by Fura-2 AM

HEK-A1 and HEK-wt cells were loaded with Fura-2 AM (c_{final} 2 µM) and stimulated with different concentrations of AITC (5 µM, 10 µM, 25 µM, 50 µM and 100 µM). DMEM was used as a negative control. The relative fluorescence signal increased with increasing AITC concentrations, suggesting hTRPA1 activation. HEK-wt cells were not affected by AITC (A). The antagonist AP18 (5 µM, 10 µM, and 25 µM) was used to confirm that these signals were hTRPA1 specific. Calcium influx was decreased and almost abolished as AP18 concentrations rose, confirming hTRPA1 correct protein folding and, as a result, functioning (B).

Figure SI 2

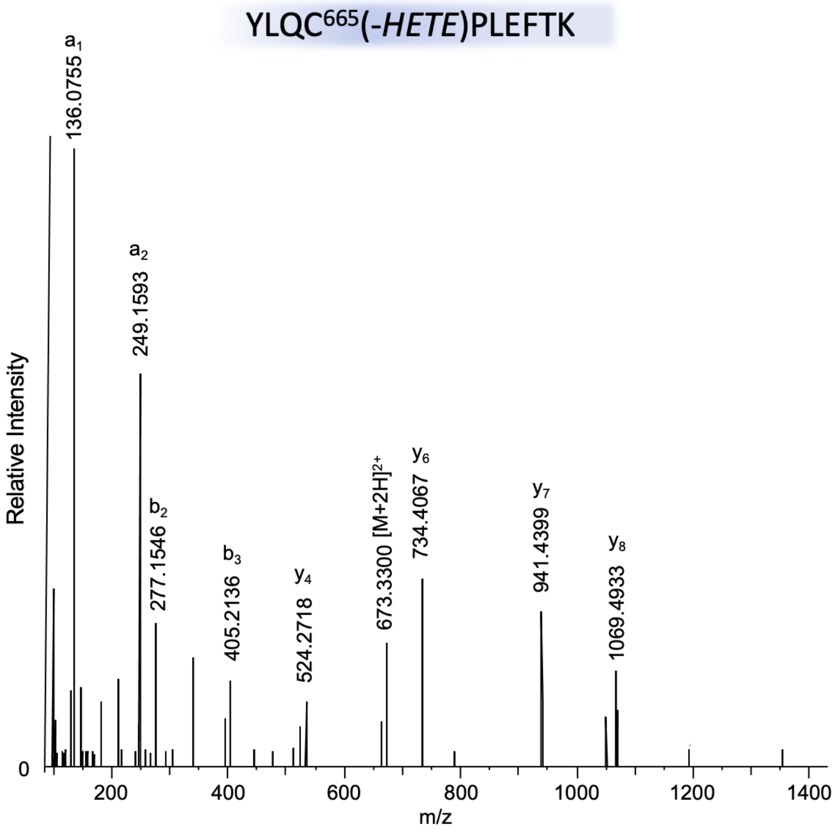

Figure SI 2: MS/HR MS spectrum of YLQC[665](-*HETE*)PLEFTK containing the alkylated Cys[665] residue

The spectrum was extracted from a μLC-ESI MS/HR MS run showing the alkylated peptide eluting at t_R 11.46 min (Fig. 3 B). The sample was obtained from HEK-A1 cells exposed to SM for 1 h followed by IMS and trypsin-cleavage. Labelled signals were assigned to product ions as indicated and are listed in detail in Table SI 1. Due to minor intensities, signals at *m/z* 105.0369 and *m/z* 137.0089 are not labelled. When compared to their theoretical mass, all ions had a mass difference typically below 5.5 ppm.

Figure SI 3

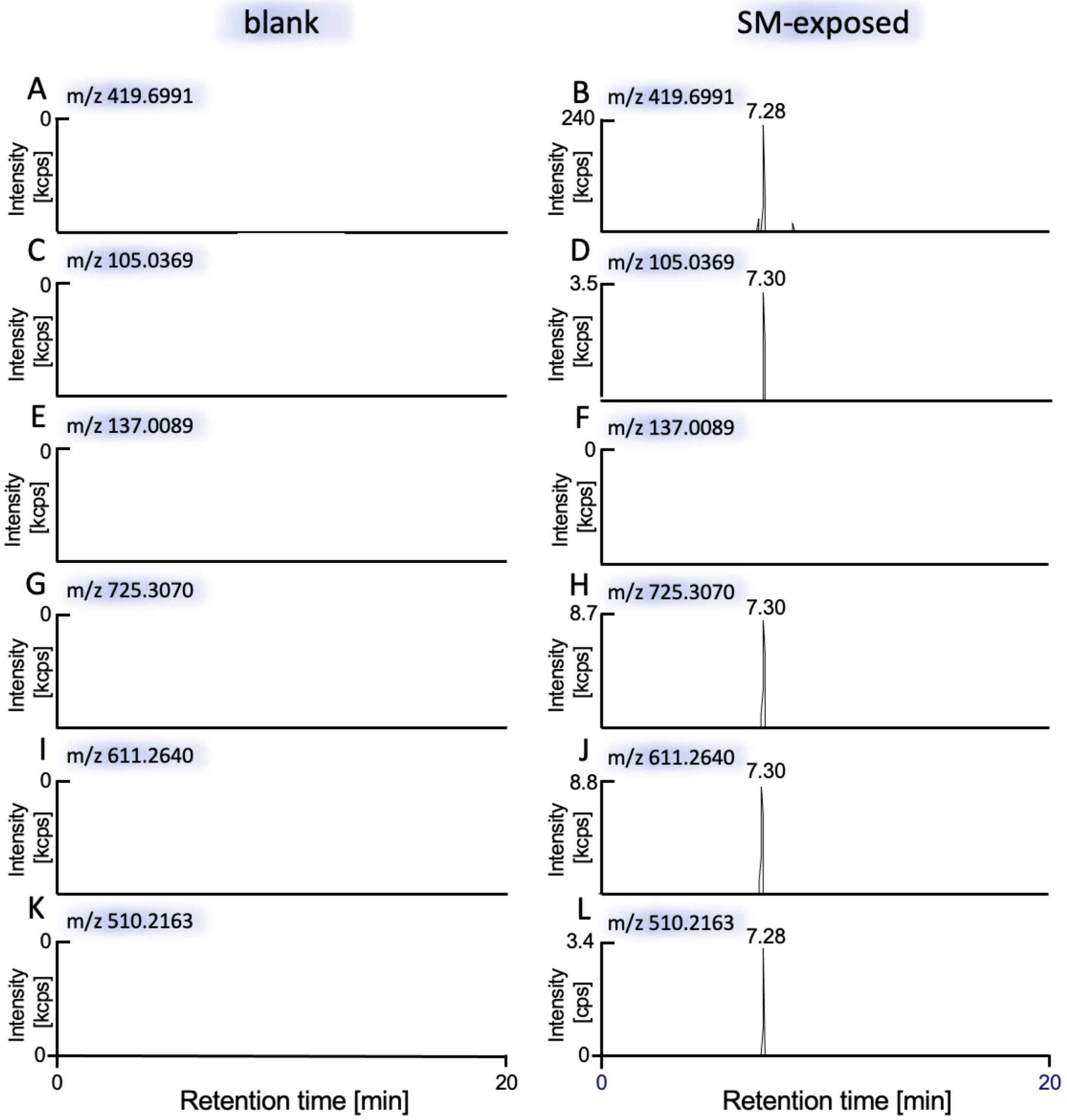

Figure SI 3: Detection of hexapeptide INTC^{462}(-*HETE*)QR $[M+2H]^{2+}$ using targeted μLC-ESI MS/HR MS (PRM)

Results from a blank (negative control) not exposed to SM are shown in left column (A, C, E, G, I, K) and results of HEK-A1 cells exposed to SM are shown in right column (B, D, F, H, J, L). Human TRPA1 was extracted from HEK-A1 cells by IMS and subjected to trypsin-mediated proteolysis. The XIC of the alkylated peptide ($[M+2H]^{2+}$ *m/z* 419.6991) is shown in part B (±3 ppm) and the XIC of diverse product ions, assigned in Table SI 2 and Figure SI 3, are shown in part D (*m/z* 105.0369), F (*m/z* 137.0089), H (*m/z* 725.3070), J (*m/z* 611.2640) and L (*m/z* 510.2163) (±10 ppm). No interferences were observed in the blank (A, C, E, G, I, K).

Figure SI 4

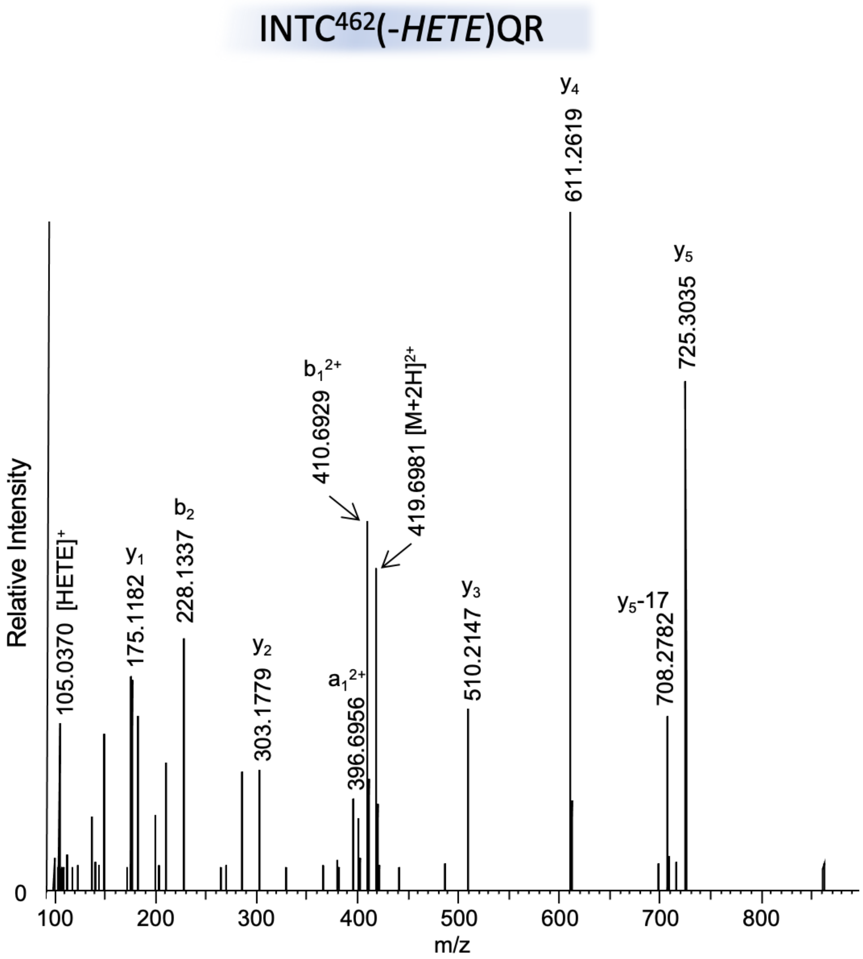

Figure SI 4: MS/HR MS spectrum of INTC^{462}(-*HETE*)QR containing the alkylated Cys^{462}

The spectrum was extracted from a µLC-ESI MS/HR MS run showing the alkylated peptide eluting at t_R 7.28 min (Fig. SI 3). The sample was obtained from HEK-A1 cells exposed to SM for 1 h followed by IMS and trypsin-cleavage. Labelled signals were assigned to product ions as indicated and listed in detail in Table SI 2. Due to minor intensity, the signal at *m/z* 137.0089 is not labelled. When compared to their theoretical mass, all ions had a mass difference typically below 5.3 ppm.

Figure SI 5

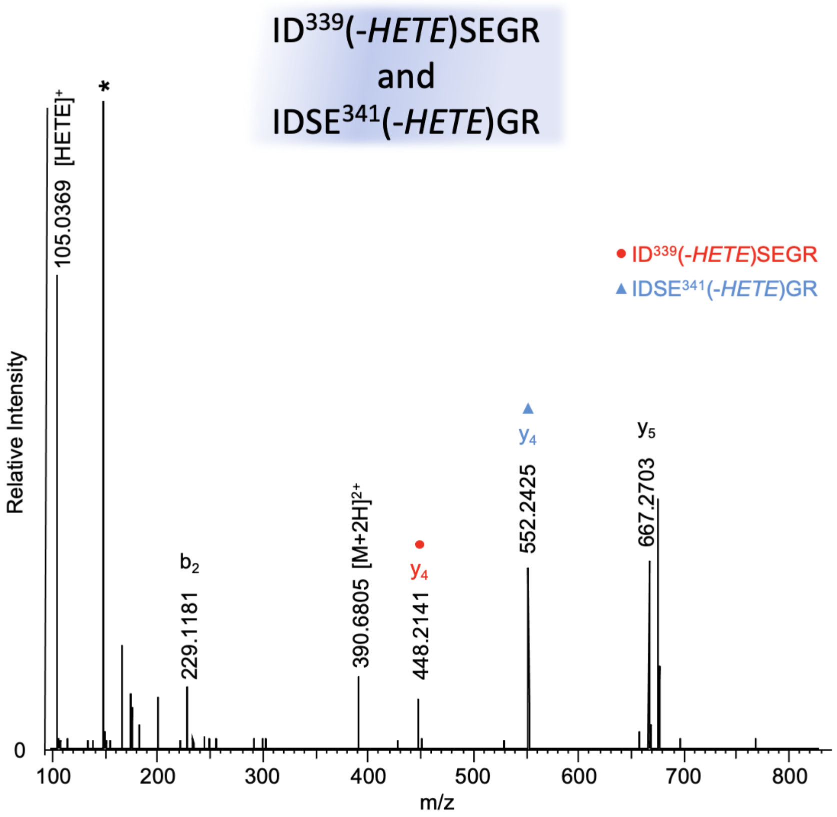

Figure SI 5: Mixed MS/HR MS spectrum of ID^{339}(-*HETE*)SEGR containing the alkylated Asp^{339} and of IDSE^{341}(-*HETE*)GR containing the alkylated Glu^{341}

Due to coelution of the adducted peptides, the mixed spectrum was extracted from a µLC-ESI MS/HR MS run showing two alkylated peptides eluting at t_R 7.49 min (Fig. 4). The sample was obtained from HEK-A1 cells exposed to SM for 1 h followed by IMS and trypsin-cleavage. Labelled signals were assigned to product ions as indicated (red circle only belonging to ID^{339}(-*HETE*)SEGR and blue triangle only to IDSE^{341}(-*HETE*)GR). When compared to their theoretical mass, all ions had a mass difference typically below 5 ppm as listed in Table SI 3 and SI 4.
